# Altered Ultrasonic Vocalization and Impaired Learning and Memory in Angelman Syndrome Mouse Model with a Large Maternal Deletion from *Ube3a* to *Gabrb3*

**DOI:** 10.1371/journal.pone.0012278

**Published:** 2010-08-20

**Authors:** Yong-hui Jiang, Yanzhen Pan, Li Zhu, Luis Landa, Jong Yoo, Corinne Spencer, Isabel Lorenzo, Murray Brilliant, Jeffrey Noebels, Arthur L. Beaudet

**Affiliations:** 1 Department of Molecular and Human Genetics, Baylor College of Medicine, Houston, Texas, United States of America; 2 Division of Medical Genetics, Department of Pediatrics and Neurobiology, Duke University School of Medicine, Durham, North Carolina, United States of America; 3 Neurogenetics Laboratory of Department of Neurology, Baylor College of Medicine, Houston, Texas, United States of America; 4 Department of Pediatrics, University of Arizona Health Science Center, Tucson, Arizona, United States of America; University of Minnesota, United States of America

## Abstract

Angelman syndrome (AS) is a neurobehavioral disorder associated with mental retardation, absence of language development, characteristic electroencephalography (EEG) abnormalities and epilepsy, happy disposition, movement or balance disorders, and autistic behaviors. The molecular defects underlying AS are heterogeneous, including large maternal deletions of chromosome 15q11–q13 (70%), paternal uniparental disomy (UPD) of chromosome 15 (5%), imprinting mutations (rare), and mutations in the E6-AP ubiquitin ligase gene *UBE3A* (15%). Although patients with *UBE3A* mutations have a wide spectrum of neurological phenotypes, their features are usually milder than AS patients with deletions of 15q11–q13. Using a chromosomal engineering strategy, we generated mutant mice with a 1.6-Mb chromosomal deletion from *Ube3a* to *Gabrb3*, which inactivated the *Ube3a* and *Gabrb3* genes and deleted the *Atp10a* gene. Homozygous deletion mutant mice died in the perinatal period due to a cleft palate resulting from the null mutation in *Gabrb3* gene. Mice with a maternal deletion (m−/p+) were viable and did not have any obvious developmental defects. Expression analysis of the maternal and paternal deletion mice confirmed that the *Ube3a* gene is maternally expressed in brain, and showed that the *Atp10a* and *Gabrb3* genes are biallelically expressed in all brain sub-regions studied. Maternal (m−/p+), but not paternal (m+/p−), deletion mice had increased spontaneous seizure activity and abnormal EEG. Extensive behavioral analyses revealed significant impairment in motor function, learning and memory tasks, and anxiety-related measures assayed in the light-dark box in maternal deletion but not paternal deletion mice. Ultrasonic vocalization (USV) recording in newborns revealed that maternal deletion pups emitted significantly more USVs than wild-type littermates. The increased USV in maternal deletion mice suggests abnormal signaling behavior between mothers and pups that may reflect abnormal communication behaviors in human AS patients. Thus, mutant mice with a maternal deletion from *Ube3a* to *Gabrb3* provide an AS mouse model that is molecularly more similar to the contiguous gene deletion form of AS in humans than mice with *Ube3a* mutation alone. These mice will be valuable for future comparative studies to mice with maternal deficiency of *Ube3a* alone.

## Introduction

Angelman syndrome (AS) is a neurodevelopmental disorder associated with severe mental retardation, absence of language development, characteristic EEG abnormalities and epilepsy, happy disposition, movement disorders, and autistic features [1]. The molecular defects underlying AS are heterogeneous. About 70% of AS patients have a large chromosomal deletion of 15q11–q13, exclusively of maternal origin. Paternal uniparental disomy (UPD) of chromosome 15 is found in ∼5% of AS patients. Mutations of the E6-AP ubiquitin ligase gene (*UBE3A*) on maternal chromosome 15 are found in about 15% of AS patients. Rarely, a microdeletion in the region designated as an imprinting center is also found in some cases of AS. The finding of loss-of-function mutations in the maternal *UBE3A* gene in patients with typical features of AS indicates that maternal deficiency for *UBE3A* is responsible for the major clinical manifestations [2], [3]. In addition, maternal, but not paternal, duplication of 15q11–q13 has been reported in about 1–2% of idiopathic autism spectrum disorder patients [4]. Recently, a small duplication covering from *ATP10A* to *GABRB3* was reported in one autism patient [5]. At the molecular level, AS patients with a large chromosomal deletion of 15q11–q13 have deficiency of the maternally expressed *UBE3A* gene and possibly of *ATP10A* in brain, and they are haploinsufficient for many other genes with biallelic expression within the 6-Mb deleted interval. These biallelically expressed genes include the GABA receptor cluster of *GABRB3*, *GABRG5*, and *GABAG3* telomeric of *UBE3A*, and *CYFIP1*, *NIPA1*, *NIPA2*, *GCG5, WHCD1L1,* and *GOLGA8E* at the centromeric end of the deletion in patients with a larger class I deletion [6], [7]. Reports have suggested that the *ATP10A* gene is maternally expressed in humans [8], [9]. One report found that *ATP10A* exhibits imprinted expression in brain but biallelic expression in non-neuronal tissues [9]. Another study reported imprinted expression of *ATP10A* in lymphoblasts as well as brains [8]. The most recent report found that *ATP10A* was expressed biallelically in brain in the majority of individuals examined while a small percentage exhibited variable monoallelic expression but not strictly maternal allele-specific. Monoallelic expression appeared to be favored by female gender [10]. Reports of imprinting status of the mouse ortholog of *ATP10A* were also not consistent. One report indicated that *Atp10a* is maternally expressed in hippocampus and olfactory bulb [11], while two other studies found that *Atp10a* is biallelically expressed in all tissues [12], [13]. Similarly, there were reports suggesting paternal, maternal, and biallelic expression of *GABRB3* in humans and mice using different experimental designs [14], [15], [16]. A recent report also suggested greatly reduced levels of the *Gabrb3* subunit in the brain of male mice with a maternally transmitted *Gabrb3* mutation but not in male mice with paternal origin of the same mutation [17].

In humans, analyses of genotype and phenotype correlation among AS patients with different classes of molecular defects have been reported [18], [19]. These analyses have revealed that AS patients with large chromosome deletions of 15q11–q13 have more severe phenotypes than AS patients with *UBE3A* mutations, paternal UPD, and imprinting mutations. In addition, there were early reports suggesting that loss-of-function *UBE3A* mutations, paternal UPD of chromosome 15, or imprinting mutations in AS were associated with relatively milder epilepsy while AS patients with a deletion of chromosome 15q11–13 had more severe epilepsy [20]. However, other studies found no genotype-phenotype correlation for EEG abnormalities and epilepsy among AS patients with different genotypes [21], [22]. One caveat of these studies was the relative small sample size which might bias the results and conclusion.

We have previously characterized an AS model with a null mutation of the *Ube3a* gene. Mice with maternal deficiency of the *Ube3a* gene recapitulated the brain-specific imprinted expression [23], [24]. Mice inheriting the *Ube3a* mutation maternally, but not paternally, displayed phenotypes that resemble AS with abnormal EEG and seizure activity, motor dysfunction, and defects in contextual fear conditioning and hippocampal long-term potentiation (LTP). These findings, together with a report by others, indicate that mice with maternal deficiency of *Ube3a* are a valid model for human AS with mutations in the *UBE3A* gene [23], [25].

Here we report generation and characterization of mutant mice with a 1.6-Mb chromosomal deletion from *Ube3a* to *Gabrb3*. The chromosomal deletion was generated by a chromosome engineering technique using cre/*loxP* and two halves of a *Hprt* (hypoxanthine-guanine phosphoribosyl transferase) minigene as described previously [26], [27], [28]. The deletion disrupted both the *Ube3a* and *Gabrb3* genes and also deleted the 1.6-Mb interval that contains the *Atp10a* gene. Analyses of maternal (m−/p+), paternal (m+/p−), and homozygous (m−/p−) deletion mice indicate that mice with a maternal deletion from *Ube3a* to *Gabrb3* provide an AS mouse model that is molecularly more similar to the common contiguous deletion form of AS, and thus valuable for future comparative studies.

## Results

### Generation of mice with a chromosomal deletion from *Ube3a* to *Gabrb3*

**Figure 1:**
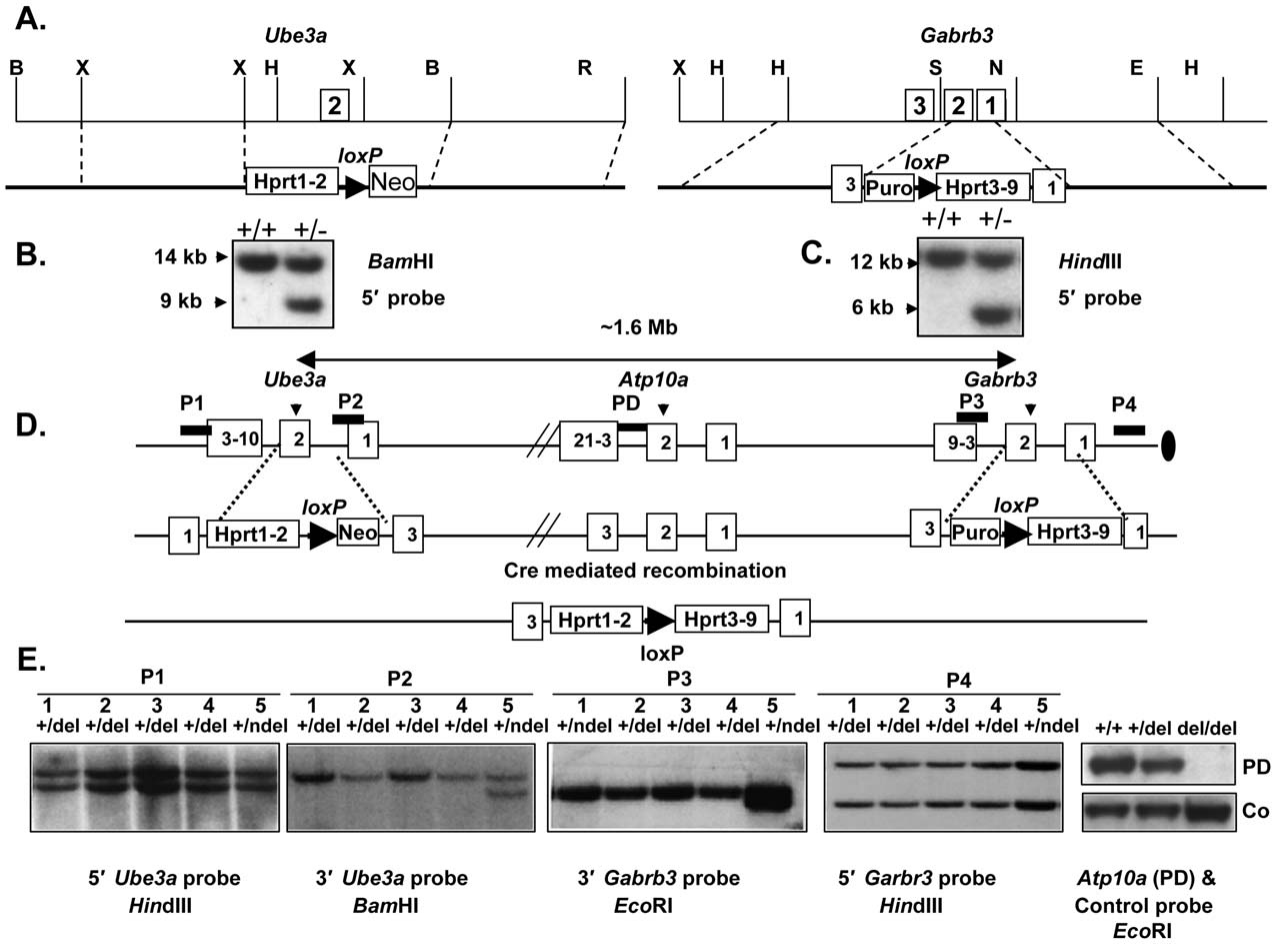
Generation of mice with a deletion from *Ube3a* to *Gabrb3* using chromosome engineering. A. Genomic maps for *Ube3a* and *Gabrb3* loci and design of targeting constructs (X, *Xba*I; H, *Hin*dIII; N, *Not*I; E, *Eco*RI; S, S*ac*II; B, *Bam*HI). The number in the rectangle indicates the exon number. B & C. Genomic DNA Southern analysis for *Ube3a* or *Gabrb3* targeted ES cell clones using 5′-flanking probes and *Bam*HI and *Hin*dIII restriction enzymes respectively. D. A genomic map from *Ube3a* to *Gabrb3* and a scheme for chromosome engineering using half of *Hprt* and Cre/*loxP* strategy. The black bars indicate genomic probes used for DNA Southern analysis. E. Confirmation of a deletion from *Ube3a* to *Gabrb3* by genomic DNA Southern analysis using probes shown in D. The probes P1 and P4 hybridize outside of the deleted interval and the probes P2, P3, and PD hybridize within the deleted interval. Four independent but indistinguishable ES cell clones (1–4, +/del) with a deletion were analyzed, and clone 5 (+ndel) was a *Ube3a* and *Gabrb3* double targeted clone without a deletion. The DNA Southern analysis using the probe PD within the *Atp10a* gene showed no signal in the DNA from homozygous deletion (del/del or m−/p− in text) mice. “Co” was a control probe from the *Snrpn* gene that is outside of deleted region in chromosome 7.

**Figure 2:**
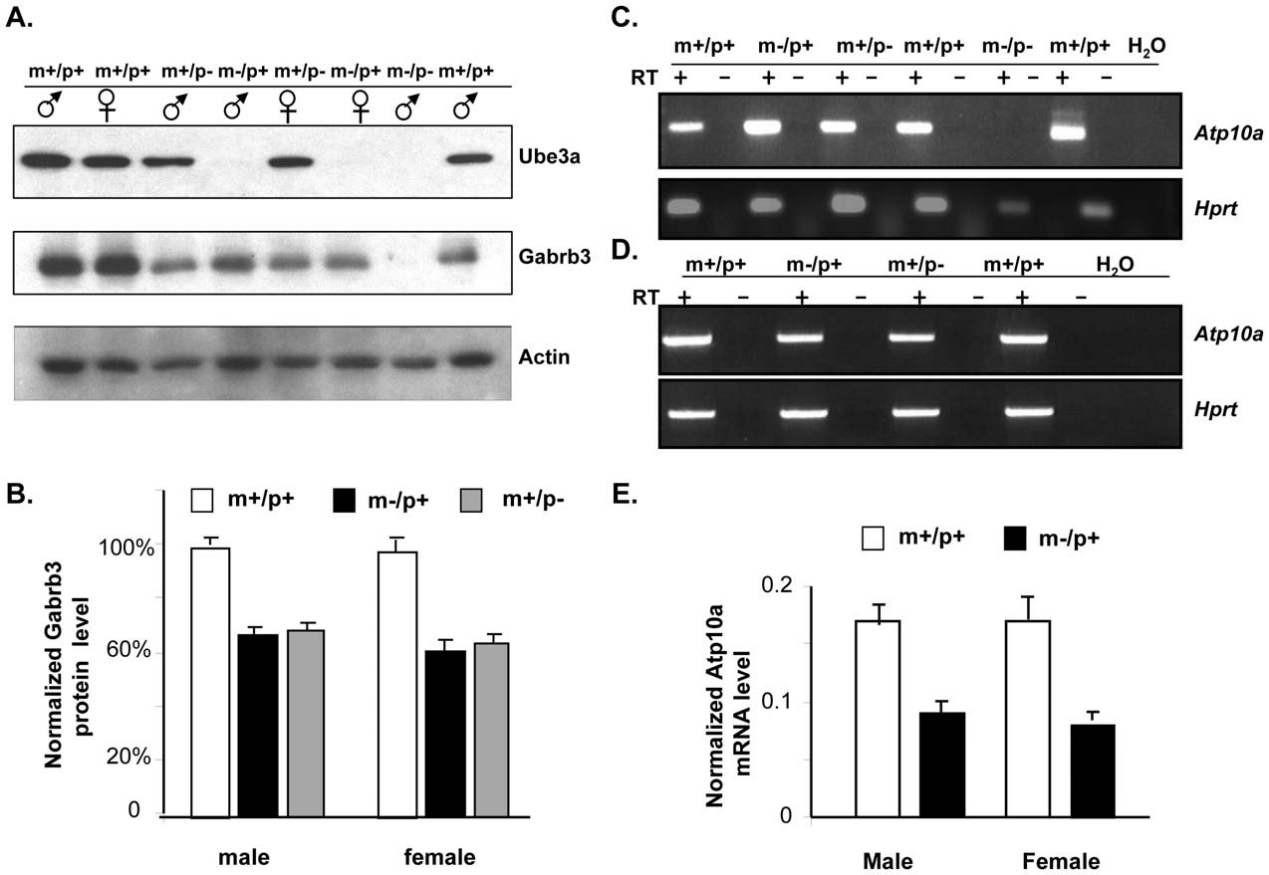
Expression analysis of *Ube3a*, *Atp10a*, and *Gabrb3*. A. Quantitative immunoblot analysis of Ube3a and Gabrb3 proteins in cerebellum. The m+/p+ is for wild-type, the m−/p+ is for maternal deletion, the m+/p− is for paternal deletion, and the m−/p− is for homozygous deletion fetus. The m+/p+ next to m−/p− was fetal brain tissue; others tissues were from adult mice. There was no detectable expression of *Ube3a* in m−/p+ and m−/p− mice indicating a null mutation in homozygotes and exclusively maternal expression of *Ube3a*. The expression of *Ube3a* in the m+/p− mice was similar to m+/p+ mice. The expression of *Gabrb3* was reduced but readily detectable in m+/p− and m−/p− mice for both males and females compared to that of m+/p+ mice. The expression level of Gabrb3 normalized to β-actin is shown in 2B. The difference between m+/p+ and m−/p+, m+/p+ and m+/p− was significant (n = 5, p<0.01) but there was no significant difference between m−/p+ and m+/p− (n = 5, p>0.05). 2C–E. Expression analysis of *Atp10a* by regular and real-time RT-PCR analyses. The labels for genotype are the same as indicated in panel A. Data from regular RT-PCR analysis for *Atp10a* using RNA isolated from cerebral cortex are shown in 2C and from the cerebellum in 2D. The expression of *Hprt* was used as a control. The absence of RT-PCR product for *Atp10a* in the m−/p− fetus indicated a null mutation of *Atp10a* in mice with a deletion. The expression of *Atp10a* was detectable and not significantly different between m−/p+ and m+/p− mice. The expression of *Atp10a* normalized to β-actin by real-time RT-PCR analysis is shown in 2E. The level of expression of *Atp10a* in m−/p+ and m+/p− was significantly lower than that of m+/p+ both in males and females (n = 3, p<0.01) but there was no significant difference between m−/p+ and m+/p− (n = 3, p>0.05).

We used the chromosomal engineering technique to generate mice with a large chromosomal deletion from *Ube3a* to *Gabrb3* as described previously [28], [29] and in detail in the Methods section. A total of three 129SvEv embryonic stem (ES) cell clones (P11A8, P11B6, and P11H8) with a deletion from *Ube3a* to *Gabrb3* were confirmed by DNA Southern analysis and injected into C57BL/6J blastocysts to obtain germline transmission. A total of 14 chimeric mice were produced. Germline transmission was obtained from ES cell clones P11A8 and P11B6 but not from P11H8. The *Gabrb3* and *Ube3a* double targeted mutations without a deletion between them were also obtained in germline (data not shown). Mice with a deletion from *Ube3a* to *Gabrb3* were identified by DNA genomic Southern analysis using genomic probes flanking and within the deletion interval as shown in the Figure 1E. Absence of signal in DNA Southern analysis using a probe hybridizing within the *Atp10a* gene indicated that the desired deletion was produced. A PCR-based mouse genotyping protocol was also developed for routine mouse colony management (data not shown). The estimated size of the deletion from *Ube3a* to *Gabrb3* was 1.6-Mb (from 64846987 bp to 66527319 bp) based on the NCBI Build 37 assembly of mouse genome sequences (www.genome.ucsc.edu). Both the *Ube3a* and *Gabrb3* genes were disrupted as shown in Figure 1D and confirmed by the RNA and protein analyses shown in Figure 2A and 2B using fetus with a homozygous deletion. Mice with a *Ube3a* single mutation have been characterized and reported previously [23]. Mice with a *Gabrb3* single mutation and *Ube3a* and *Gabrb3* double mutations produced from this experiment have not been fully characterized. However, *Gabrb3* mutant mice with a different mutation have been reported previously by others [30]. Because of involvement of imprinting in the region, a specific breeding scheme diagramed in supplemental Figure S4 was followed to produce mice for experiments described below.

### Expression analysis of *Ube3a, Atp10a*, and *Gabrb3* in mice with a deletion from *Ube3a* to *Gabrb3*

We examined the parental origin of expression for *Ube3a*, *Gabrb3*, and *Atp10a* in mice inheriting a paternal or maternal deletion by RNA or protein analysis. Tissues from homozygous deletion mice (m−/p−) were included in the analysis as controls. The expression of *Gabrb3* and *Ube3a* was examined by quantitative immunoblot analysis while the expression of *Atp10a* was performed by regular and real-time RT-PCR analysis because a specific antibody against *Atp10a* was not available. Because of a report of gender differences in monoallelic expression of *Gabrb3* in mice and *ATP10A* in humans [10], [17], we performed the expression analysis in both sexes of mice with a deletion from *Ube3a* to *Gabrb3*. Different brain regions, including hippocampus, cerebellum, and cerebral cortex, were dissected and compared to liver tissues to determine if there were tissue-specific differences. The data from all three brain regions were similar and only data from cerebellum are shown. As shown in Figure 2A, the protein level of *Ube3a* in m−/p+ mice was undetectable in cerebellum while the protein level of *Ube3a* in m+/p− mice was comparable to that in m+/p+ mice. The expression in the cerebral cortex of m−/p+ mice was similar to cerebellum and almost exclusively maternal (data not shown). The expression of *Ube3a* was readily detectable and similar in liver tissues among m−/p+, m+/p−, and m+/p+ mice which indicated biallelic expression of *Ube3a* in non-CNS tissues (data not shown). These data are consistent with the previous reports that *Ube3a* is maternally expressed in brain [23], [24] but the expression in the cerebral cortex was slightly different from the reports by RNA *in situ* in UPD and *Ube3a* mutant mice [23], [31]. The expression of *Gabrb3* was also readily detectable in m−/p+, m+/p−, and m+/p+ mice in different brain regions in both males and females by quantitative immunoblot analysis as shown in Figure 2A. The level of Gabrb3 was 61.6±2.3% of wild-type in the m−/p+ mice and 62.5±2.1% of wild type in the m+/p− mice for both sexes together which is slightly higher than 50% as predicted for heterozygotes. The level of Gabrb3 in m+/p− and m−/p+ mice for males and females normalized to m+/p+ separately is summarized in Figure 2B. There was no significant difference between m+/p− and m−/p+ for males (n = 5, p = 0.75) or females (n = 5 and p = 0.29). There was no evidence for parental origin effect on the expression of *Gabrb3* in the tissues examined. Similarly, expression of *Atp10a* was readily detectable by regular RT-PCR analysis in the cerebellum, cerebral cortex, hippocampus, and liver tissues of m−/p+ and m+/p− mice (Figure 2C and 2D, and data not shown for hippocampus and liver tissues). Among 8 (4 males and 4 females) m−/p+ and 7 m+/p− mice (4 females and 3 males) analyzed by RT-PCR, we did not observe the exclusively monoallelic expression of *Atp10a* in the cerebellum and hippocampus that was described in human brains [10]. To examine whether there was a quantitative or gender specific imprinting expression of *Atp10a*, we also performed real-time RT-PCR analysis for *Atp10a* in cerebellar tissues from m−/p+ and m+/p+ mice of both sexes. Expression of *Atp10a* normalized to the level of *Gadph* was 51.8±0.8% of wild type in m−/p+ male mice and 50.6±0.4% of wild type in m−/p+ male mice. There was no significant difference in the expression of *Atp10a* between male and female m−/p+ mice (p = 0.45, n = 3). These results indicate that the *Atp10a* gene is biallelically expressed in the tissues examined although the possibility of cell-type specific or developmental stage specific imprinting cannot be ruled out.

### Perinatal lethality of mice with homozygous deletions from *Ube3a* to *Gabrb3*

**Figure 3:**
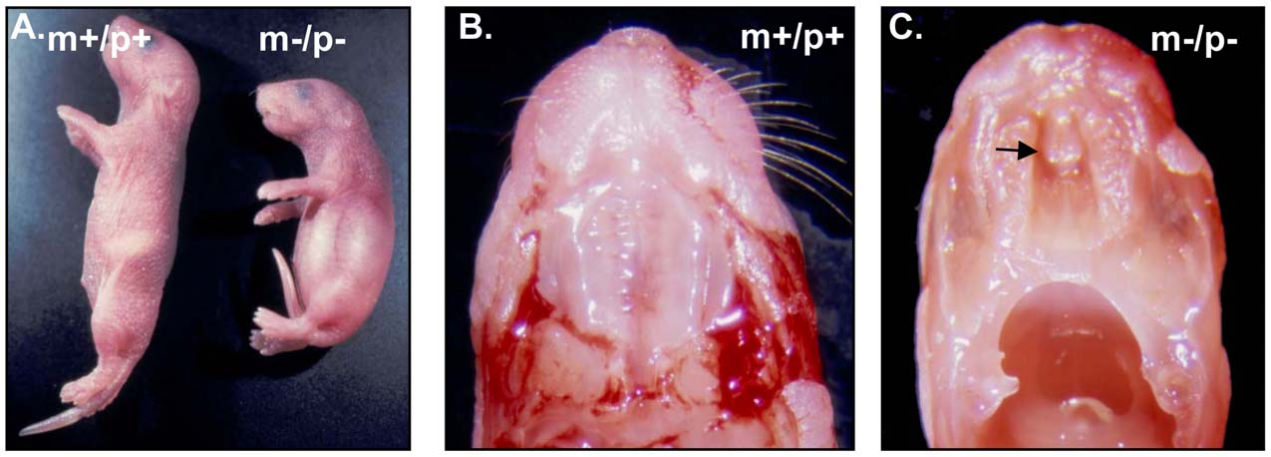
Perinatal lethality and cleft palate in newborn mice with a homozygous deletion from *Ube3a* to *Gabrb3* (m−/p−). A. Growth retardation of 2-day-old pup with a homozygous deletion from *Ube3a* to *Gabrb3*. B & C. Cleft palate in a pup with a homozygous deletion from *Ube3a* to *Gabrb3*. The black arrow points to the cleft palate in pup with a homozygous deletion (m−/p−).

Heterozygous mice inheriting a deletion of either paternal or maternal origin did not have any developmental defect. At 3 months of age, there was no difference in weight between m−/p+ (22.0±0.8 g) and m+/p+ mice (22.6±0.7 g) (p = 0.55), or between m+/p− (20.8±0.9 g) and m+/p+ mice (20.9±0.8 g) (p = 0.9). There was no apparent difference between m−/p+ and m+/p− mice by visual inspection. The lifespan of m−/p+ and m+/p− mice was similar to their m+/p+ littermates. Homozygous mutant mice with a deletion from *Ube3a* to *Gabrb3* (m−/p−) were smaller at birth and most died within 1 to 3 days after birth due to a cleft palate and poor feeding (Figure 3A). At the weaning age, there was a significantly skewed distribution of genotypes from Mendelian inheritance (wild-type 20; heterozygotes 39; and homozygotes 2, p<0.001) because of perinatal lethality. We carefully examined each pup (n = 12) that died perinatally. Nine of 12 dead pups had a cleft palate, a representative example of which is shown in Figure 3C. No other obvious structural defects were found in pups with a homozygous deletion from *Ube3a* to *Gabrb3*. Functional defects such as poor feeding may be considered as the cause of perinatal death for pups without a cleft palate. Because it was reported previously that a homozygous null mutation of *Gabrb3* caused cleft palate [30], we also generated mice with single homozygous *Gabrb3* mutation for the purpose of comparison. A cleft palate was found in 6 out of 7 pups with a homozygous *Gabrb3* mutation. Two mice with a homozygous deletion from *Ube3a* to *Gabrb3* survived beyond the perinatal period but died at 24 and 28 days of age without a known cause. These two mice were ∼20% of smaller than their wild type littermates and without an apparent cleft palate. To determine whether embryonic lethality was associated with m−/p− mice, a total of 19 pups from three litters were dissected and genotyped at 16 days of gestational age. The distribution of genotypes was not significantly different from the expected Mendelian inheritance (wild-type 5; heterozygotes 10; and homozygotes 4, p>0.05). These data suggested that a homozygous deletion from *Ube3a* to *Gabrb3* did not cause embryonic lethality and that the function of *Atp10a* is probably not critical for embryonic development.

### Spontaneous seizures and cortical discharges in mice with a maternal deletion from *Ube3a* to *Gabrb3*

**Figure 4:**
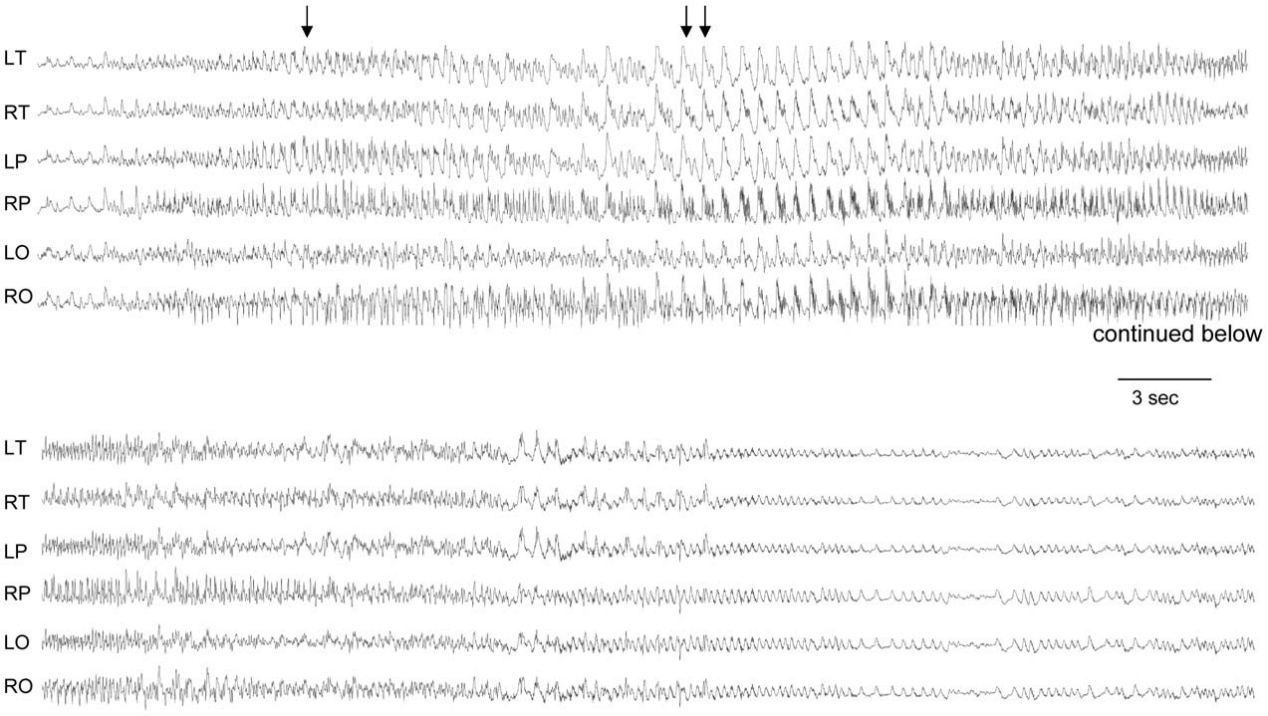
Abnormal EEG in mice with a maternal deletion from *Ube3a* to *Gabrb3* (m−/p+). Multielectrode continuous EEG tracing shows representative generalized rapid cortical spike seizure discharge in freely moving m−/p+ deletion mice. Top-bottom: temporal (T), parietal (P), and occipital leads (O) alternating between left (L) and right hemisphere (R) indicated as LT, RT, LP, RP, LO, and RO. The six panels in the top tracing were continued in the lower section. Single arrow indicates the onset of sudden isolated giant spike-wave discharges with no behavioral accompaniment, and double arrow marks progression to higher spike discharge frequencies.

Spontaneous visible tonic-clonic seizures were observed in 21.4% (6/28) of m−/p+ mice at 3–6 months of age but not in m+/p+ mice (n = 32) or m+/p− mice (n = 36) on the relatively homogenous C57BL/6J genetic background after more than 6 generations of backcrossing to C57BL/6J mice. The frequency of seizure activity in Ube3a to Gabrb3 maternal deletion mice was not different from that of maternal Ube3a deficiency alone (21%) previously reported [23]. To further characterize seizure activity, we performed chronic electroencephalographic (EEG) recordings for 3 m−/p+, 3 m+/p−, and 3 m+/p+ mice at 3–4 months of age. As shown in Figure 4, EEG in m−/p+ mice showed a pattern beginning with sudden isolated giant spike-wave discharges with no behavioral accompaniment, but then progressed to higher spike discharge frequencies, at which point the mice displayed sustained behavioral arrest and subtle myoclonic movements. These seizures lacked any tonic posturing or postictal phenomenology, and the mice rapidly resumed normal behavior (a sample video of recording is included in the supplementary data, Video S1). Frequent generalized cortical single interictal spike discharges were also present in m−/p+ mice (not shown). We also performed EEG recording in maternal Ube3a deficiency alone mice, and consistent with previous findings [23], an abnormal EEG was observed (data not shown). Electrographic seizures or discharges were not observed in m+/p− and m+/p+ mice during the recording.

### Behavioral analyses

We conducted a battery of behavioral tests for mice with a maternal or paternal deletion from *Ube3a* to *Gabrb3*. Mice used for behavioral tests were backcrossed to the C57BL/6J background for more than 6 generations as described in Methods section and diagramed in supplementary Figure S4. Behavioral tests included the following: open field, light dark, rotarod, prepulse inhibition (PPI), fear conditioning, Morris water maze, hot plate, and ultrasonic vocalization (USV) recording. Mice at age 8–10 weeks with a maternal deletion (m−/p+), paternal deletion (m+/p−), and their wild type (m+/p+) littermates were used for the tests. A total of 10 m−/p+ mice (5 males and 5 females) were compared to a total of 16 m+/p+ (7 males and 9 females), and 11 m+/p− mice (6 males and 5 females) were compared to 10 m+/p+ (5 males and 5 females). For USV recording, newborn pups of 6 day old were used for all genotypes. A total number of 18 m−/p+ pups (8 males and 10 females) and 17 m+/p+ (7 males and 10 females); and 27 m+/p− pups (12 males and 15 females) and 22 m+/p+ (12 males and 10 females) were analyzed. Mice used for USV recording were not used for other behavioral tests. Experimenters were blinded to the genotype during testing, and tested mice were re-genotyped after completion of testing. There was no significant difference between males and females for these tests. Therefore, the results were pooled for each test and described below.

### Locomotor activity in the open-field

The open-field test was used to assess exploratory activity and anxiety-related responses in a novel arena. The travel distance during each testing block was similar for m−/p+ and m+/p− mice and their m+/p+ littermates (p = 0.12 for m−/p+ and p = 0.23 for m+/p−) (Figure S1A and S1B). There was no significant difference in total travel distance between m−/p+ mice and their m+/p+ littermates (Figure S1C; p = 0.4), and there were no differences in the center and total distance ratio (data not shown). Similarly, there was no difference in total travel distance between m+/p− mice and their m+/p+ littermates (Figure S1C, p = 0.42).

### Light-dark exploration

**Figure 5:**
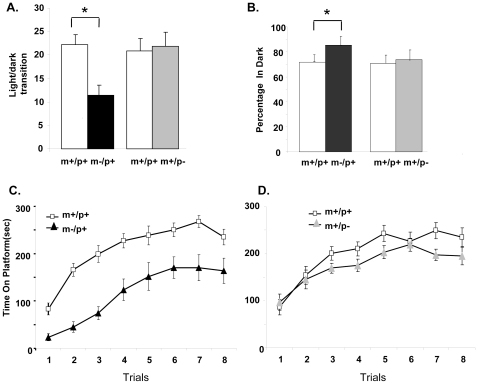
Abnormal light-dark exploration and impaired rotarod performance in mice with a maternal deletion from *Ube3a* to *Gabrb3* (m−/p+) but not a paternal deletion (m+/p−). A & B. Light-dark transition. The m−/p+ but not m+/p− mice showed a significantly lower number of light and dark transitions, shown in A (p = 0.003), and also spent significantly longer time in the dark chamber, shown in B (p = 0.008). There was no difference between m+/p+ of maternal and paternal group for both light-dark transition and percentage of time stayed in dark. C & D. Rotarod performance. The time that m−/p+ mice remained on the accelerating rotarod was significantly shorter than m+/p+ littermates for all trials, shown in C (p<0.0001). The overall performance of m+/p− mice was slightly worse than that of m+/p+ littermates, but the difference was not statistically significant, shown in D (p>0.05). * p<0.05.

The light–dark exploration test was also used to assess anxiety-related responses. The number of light-dark transitions was significantly lower in m−/p+ mice than in m+/p+ littermates (p = 0.003) but not significantly different between m+/p− mice and m+/p+ littermates (p = 0.8) (Figure 5A). In addition, m−/p+ mice stayed for a significantly longer time in the dark chamber than their m+/p+ littermates (p = 0.008) (Figure 5B). There was no significant difference for light dark transition (p = 0.25) and for the percentage of time stayed in dark (p = 0.32) between m+/p+ of maternal and paternal groups. But the difference between m−/p+ and m+/p− was significant (p = 0.006). These results suggest anxiety related behavior assayed by this task in m−/p+ mice when compared to their m+/p+ littermates.

### Rotarod test

There was no apparently abnormal gaits and weakness of limbs in the maternal and paternal deletion mice. We then used rotarod to study motor coordination and motor learning skills. As shown in Figure 5C, m−/p+ mice performed significantly worse on an accelerating rotarod than their m+/p+ littermates (p<0.0001). The performance of m+/p− mice was slightly worse than their m+/p+ littermates but the results were not statistically significant (Figure 5D, p = 0.06).

### Hotplate

The hotplate test is an indicator of the sensitivity of animals to painful stimuli. The time to the first hind-limb response is shown in the Figure S1D. There was no significant difference between m−/p+ mice and m+/p+ littermates (p = 0.9). Similarly, there was no difference between m+/p− mice and their m+/p+ littermates (p = 0.46).

### Prepulse inhibition (PPI)

As shown in Figure S2, there was no difference in maximal startle response between m−/p+ mice and their m+/p+ littermates or between m+/p− mice and their m+/p+ littermates. There was a trend towards increased PPI in m−/p+ mice, but the difference was not statistically significant (Figure S2B, p = 0.17). There was no significant difference between m+/p− mice and their m+/p+ littermates (Figure S2C; p = 0.75).

### Impaired contextual fear learning in maternal deletion mice

**Figure 6:**
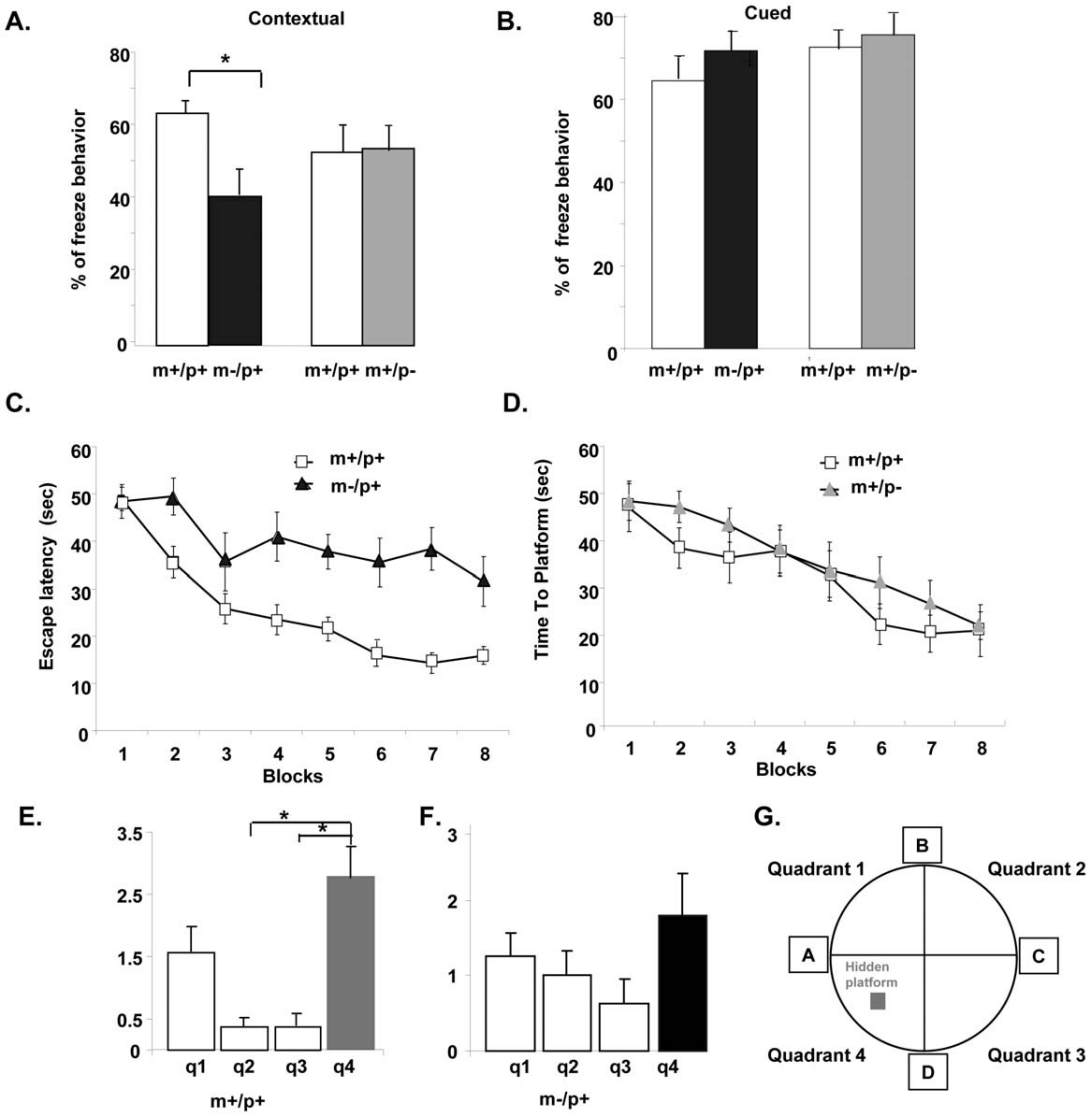
Impaired learning and memory task in mice with a maternal deletion from *Ube3a* to *Gabrb3* (m−/p+). A & B. Fear conditioning. The m−/p+ but not m+/p− mice showed significantly less freezing behavior in contextual fear conditioning (A) but not for cued fear conditioning. C to G. Morris water maze task. The m−/p+ but not m+/p− mice showed significantly longer escape latency than m+/p+ littermates (p<0.001 for m−/p+ and p>0.05 for m+/p−). In the probe trial, analysis of platform crossing found that m+/p+ mice displayed a significantly biased searching strategy, shown in E (p<0.001), while m−/p+ mice displayed a random searching strategy, shown in F (p>0.05). Post hoc analysis of m+/p+ mice revealed that the difference was significant only between quadrant 4 and 2 (p<0.01), and quadrant 4 and 3 (p<0.02). The labeling of each quadrant and location of the hidden platform is illustrated in G. * p<0.05.

Levels of freezing behavior for the contextual and auditory cued conditioning fear tests are shown in Figure 6; m−/p+ mice displayed a significantly lower level of freezing behavior in contextual fear conditioning than their m+/p+ littermates (p = 0.03). A significant difference was not observed between m+/p− and m+/p+ mice for contextual fear conditioning (p = 0.59) (Figure 6A). There was no significant difference in cued fear conditioning for either m−/p+ (p = 0.31) or m+/p− (p = 0.58) mice compared to that of their m+/p+ littermates (Figure 6B). These findings suggest that m−/p+ mice, but not m+/p− mice, have a selective impairment in fear conditioning that is associated with the context or environment where the shock occurred but not in cued fear conditioning.

### Spatial learning in the Morris water maze task

We performed the Morris water maze test to evaluate spatial learning and memory. As shown in Fig. 6C and 6D, both m−/p+ and m+/p− mice had very similar performances on day 1 of training which indicated the ability of swimming was comparable. However, m−/p+ mice, but not m+/p− deletion mice, took a significantly longer time to locate the hidden platform in subsequent trials (p<0.001 for m−/p+ and p = 0.8 for m+/p− mice). Probe trials were performed after the last trial to determine if a different strategy of searching for the hidden platform was used by mice with different genotypes. As shown in Figure 6E and 6F, m+/p+ mice displayed a spatially biased search pattern by making significantly more platform crossings in the target quadrant 4 (p<0.001); a similar spatially biased searching pattern was not observed in the m−/p+ mice (p = 0.58). Post hoc analysis for m+/p+ mice showed that the difference was significant only between quadrant 3 and quadrant 4 (p<0.02), and between quadrant 2 and quadrant 4 (p<0.01) (see Figure 6G). The m+/p− mice showed a searching pattern similar to m+/p+ mice (data not shown).

### Ultrasonic vocalizations (USVs)

**Figure 7:**
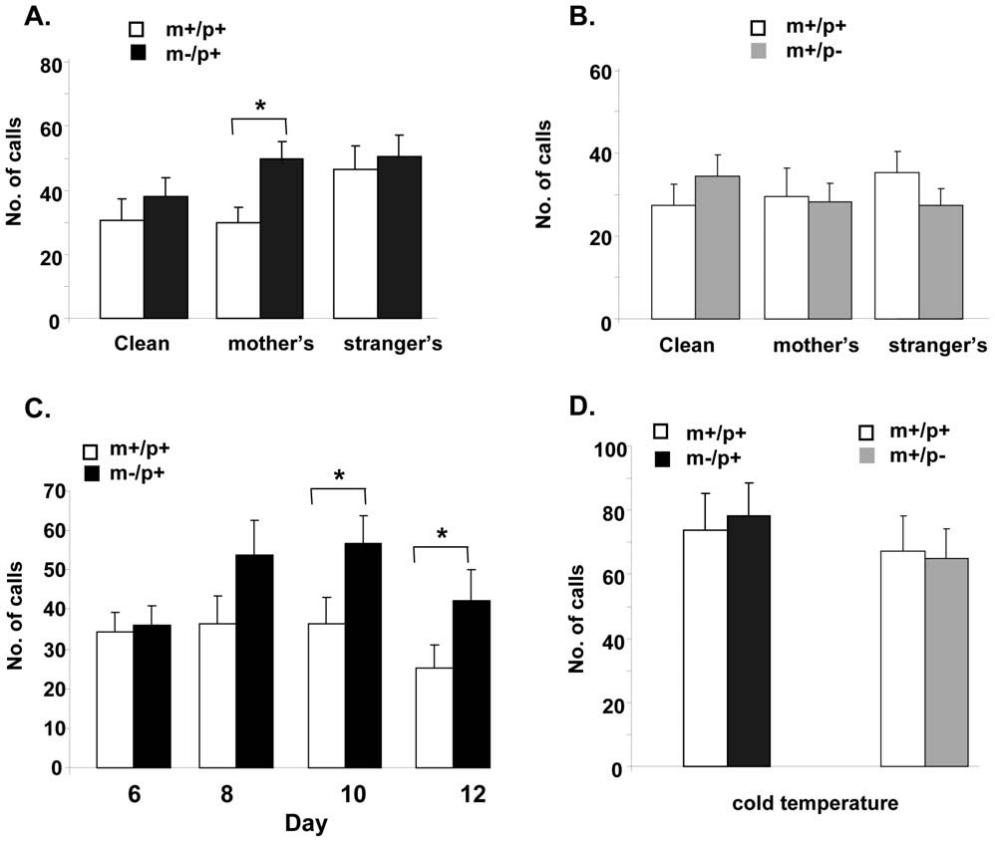
Increased ultrasonic vocalizations (USVs) of newborn pups with a maternal deletion (m−/p+) from *Ube3a* to *Gabrb3*. A and B. Comparison of m−/p+ and m+/p− pups respectively to their m+/p+ littermates in different beddings. The m−/p+ pups emitted significantly more USVs than that of m+/p+ littermates at mother's bedding (p = 0.006) (2B) but not for m+/p− pups(2C, p>0.05). C. Comparison of USVs of m−/p+ to m+/p+ at different postnatal days. The m−/p+ pups emitted more USVs overall than m+/p+ mice but this was only statistically significantly on day 10 (p = 0.02) and day 12 (p = 0.05). The three-way (genotype X bedding X day) with mixed design ANOVA did not reveal significant interaction among them (p = 0.07). Similarly, two-way ANOVA did not reveal significant interactions between genotype and bedding (p = 0.15), and between genotype and day (p = 0.29). D. Comparison of USVs for m−/p+ and m+/p− pups recorded in clean bedding at low temperature (4–6°C) on day 10 and day 12. There was no significant difference between m−/p+ and their m+/p+ littermates (p = 0.82), or between m+/p− and their m+/p+ littermates (p = 0.3). * p<0.05.

We recorded ultrasonic vocalizations (USVs) for pups at age of postnatal day 6, 8, 10, and 12 on three different beddings of clean, mother's, and stranger's. The number of USVs recorded during a period of five minutes for each day on different beddings was analyzed separately (Figure S3) and together (Figure 7). When data were analyzed separately on different days and beddings, it was noted that the USV production of m−/p+ pups was lower on day 6 of the clean bedding than other beddings but not statistically significant (p = 0.07). However, the USV production of m−/p+ pups increased on subsequent days and was significantly higher (p = 0.034) on day 12 when compared to those of m+/p+ pups (Figure S3). When the data on different beddings were analyzed regardless of days, there was an overall trend of increased USVs in m−/p+ pups compared to those of m+/p+ littermates in different beddings. The difference was statistically significant in the group recorded on the mother's bedding (p = 0.006), borderline in the clean bedding group (p = 0.046), but not significant in the stranger's bedding (p = 0.12) (Figure 7A). There were no significant differences between m+/p− and m+/p+ pups in different beddings (p = 0.25) (Figure 7B). When the data were analyzed on the different days regardless of beddings, there were significantly increased USVs in m−/p+ pups compared to their m+/p+ littermates (p = 0.011). Post hoc analysis revealed that the difference between m−/p+ and m+/p+ littermates was significant on day 10 (p = 0.02), borderline on day 12 (p = 0.05), but not significant on day 6 (p = 0.81). The three-way (genotype *vs* bedding *vs* day) mixed design ANOVA did not reveal significant interaction among them (p = 0.07). Similarly, two-way ANOVA did not reveal significant interaction between genotype and bedding (p = 0.15), and genotype and day (p = 0.29). There was no difference in USV production between m−/p+ and m+/p+ pups (p = 0.82), or between m+/p− and m+/p+ pups (p = 0.3) recorded on clean bedding in cold temperature on day 10 and day 12. These data indicated a normal stress response and capability to produce the USVs for both m−/p+ and m+/p− pups. Thus, the abnormal pattern of USV production observed in maternal deletion pups may suggest abnormal signaling or communication behaviors between mother and offspring.

## Discussion

### Application of chromosome engineering strategy to model the human chromosome microdeletion syndromes

Using Cre/*loxP* and *Hprt* minigene chromosome engineering strategies, we have successfully generated and characterized mutant mice with a 1.6-Mb chromosomal deletion in mouse chromosome 7 corresponding to the critical region homologous to that deleted in human Angelman syndrome. With the application of array CGH (comparative genomic hybridization) in clinical genetics practice, an increasing number of new microdeletion syndromes are being characterized [32], [33], [34]. Together with several examples reported previously by us and others [28], [35], this report emphasizes the value of this strategy in dissecting the pathogenesis of human microdeletion syndromes using mouse models [29]. We have observed a workable frequency of Cre mediated recombination between two *loxP* sites that were 1.6-Mb apart, and others have reported similar frequencies in other chromosomal regions [36]. The challenge of this strategy is probably still related to the difficulty of obtaining germline transmission for targeted ES cells that are subjected to multiple electroporations during the procedure [28], [37], [38].

### No evidence for allele-specific expression of the *Atp10a* and *Gabrb3* genes in mice

We have shown that there was no detectable expression of *Ube3a*, *Atp10A*, and *Gabrb3* in homozygous deletion mice by RT-PCR or immunoblot analysis, which indicated that the deletion resulted in null alleles for all three genes. The expression pattern of *Ube3a* in the cerebellum and hippocampus in maternal deletion mice was consistent with previous reports of exclusive maternal expression of *Ube3a* in these regions [23], [24]. We also observed that the expression in the cerebral cortex was almost exclusively maternal. This is slightly different from the previous report that the expression of *Ube3a* was predominantly maternal and region specific in the cerebral cortex by RNA in situ in UPD mice [23], [31]. The discrepancy could be due to the sensitivity of different techniques or different mutations analyzed. Using mice carrying a paternal or maternal deletion of *Atp10a* and *Gabrb3*, we were able to examine the parental origin of expression for *Atp10a* and *Gabrb3* directly. Our analysis did not provide any evidence supporting exclusive maternal expression of *Atp10a* but indicated biallelic expression of *Atp10a* in the brain regions of mice examined by quantitative RNA expression analysis. This analysis also did not reveal monoallelic expression of *Atp10a* that was reported in a small fraction of human brains and associated with gender [10]. This is in agreement with two studies that found no imprinted expression in any tissue examined [12], [13] but differs from a third report suggesting predominant maternal expression of *Atp10a* in hippocampus and olfactory bulb in mice [11]. It is not immediately clear why the results from these studies are different. One possibility is the effect of mouse genetic background on imprinting, but this has not been fully investigated [39]. Our expression analysis of *Atp10a* was performed in mice with a relatively homogenous C57BL/6J genetic background that overlaps with, but is not the same as that used in the other studies. The technical limitations of the different methods used should also be considered. Our strategy of using *Atp10a* knockout mice was different from the other studies, which used strain-specific single nucleotide polymorphisms to determine the parental origin of expression, or studied in PWS imprinting center mutant mice. A limitation of our analysis is that we could not rule out a cell type-specific imprinting expression of *Atp10a*. In the future, expression analysis by RNA *in situ* hybridization or immunohistochemistry may help to determine if cell type-specific imprinting is associated with *Atp10a* expression. Similarly, our analysis did not reveal any evidence for a parental origin of expression for *Gabrb3* but again the possibility of cell type or developmental stage specific expression cannot be completely ruled out.

### The function of *Atp10a* gene

The function of *Atp10a* in mammals is unknown. Homozygous mice with a deletion from *Ube3a* to *Gabrb3* died 1 to 3 days after birth and did not have any increased embryonic lethality or additional structural defects compared to mice with a null mutation of either *Gabrb3* or *Ube3a* alone, or *Gabrb3* and *Ube3a* double mutation without a deletion (Jiang and Beaudet, unpublished data). These observations suggest that the function of *Atp10a* is probably not essential during embryonic development. Studies of mice carrying a radiation-induced large deletion proximal to the 3′-end of *Snrpn* to the *p* locus that included *Atp10a*, indicated that *Atp10a* may have an important role in insulin resistance and glucose metabolism [40], [41]. However, no direct conclusion regarding the role of *Atp10a* could be made because the deletion is large and mice are haploinsufficient for many genes in addition to *Atp10a*. It would be interesting to perform the experiment described by Dhar et al. [40] to determine whether insulin resistance can also be found in mice with the *Ube3a* to *Gabrb3* deletion. Additional studies of homozygous deletion mice and a careful comparison to mice with null mutations of *Ube3a* or *Gabrb3* in the region may reveal the function of *Atp10a*, or it may be necessary to produce an *Atp10a* single knockout in order to ultimately understand the function of this gene.

### Increased USVs in the maternal deletion mice

Ultrasonic vocalizations (USVs) in neonatal mice have been studied both as early communicative behavior between pups and mothers, and as a sign of response to environmental stress such as separation and cold temperature [42]. Recording of USVs has been increasingly used as a tool to analyze behavioral phenotypes of mouse models of neurodevelopmental disorders although interpretation of the findings remains a subject of debate [43]. Currently, there is no standardized protocol for recording USVs in the literature [44]. We followed a previously described protocol with minor modifications [45]. The rationale for recording USVs on different beddings was based on the fact that different odors may present important cues for social stress or isolation [42]. Recording in cold temperature was designed to evaluate the response to a known stress for USV production [46]. The protocol of recording on different days was based on the observation that the peak of USV production varied on different postnatal days and differed among different mouse genetic backgrounds [47]. From reviewing the limited reports of USVs recording in knockout mice in the literature, we anticipated the possibility that m−/p+ pups might have reduced USVs because of absence of language and autistic features in AS. Thus the finding of an overall increase in USVs in pups with a maternal deletion from *Ube3a* to *Gabrb3* was somewhat unexpected but not surprised. The comparable rate of USVs between m+/p+ and m−/p+ mice at cold temperature suggested that m−/p+ pups had a normal response to cold stress. The significantly increased production of USVs in different beddings and on different days for m−/p+ pups may indicate increased anxiety and response to social isolation stress. However, this interpretation seems counterintuitive because the difference between m+/p+ and m−/p+ was most significant in the mother's bedding which was intended to be less stressful because of the maternal odor. Alternatively, the significant difference in USV production between m+/p+ and m−/p+ pups recorded in the mother's bedding may reflect impairment related to maternal attachment behaviors between mother and offspring. In the earlier reports, the characteristic happy disposition and friendly behaviors in AS patients were considered to be contextually inappropriate and not related to the environment stimuli [18]. It has also been reported that AS patients have significant autistic features and often meet the diagnosis of autism spectrum disorder through autism diagnostic evaluation [48], [49]. In contrast, other recent behavioral studies suggest that happy disposition like smiling and laughing behaviors in AS were influenced by environmental clues and different social settings [50], [51]. By carefully observing social behaviors among children with AS caused by a deletion of 15q11–q13, Oliver et al. reported that AS patients smiled more and evoked a higher level of adult attention than the control group in different settings [51]. They concluded that the excessive smiling and laughing behaviors seen in AS patients may indicate an increase in signaling behaviors. Thus, the observation of increased production of USVs in m−/p+ pups may suggest that increased USVs is analogous to the increased signaling behaviors observed in human AS. It should be noted that the human AS behavioral observation studies by Oliver et al. were carried out only in a small number of AS patients, all with a deletion of 15q11–q13 [51], [52]. An interesting question is whether AS patients with mutations in the *UBE3A* gene have the same behavioral profile related to smiling and laughing as those with a deletion of 15q11–q13. With the availability of AS mouse models with *Ube3a* mutation alone and a deletion from *Ube3a* to *Gabrb3* in this report, a similar question can also be addressed in mice by recording USVs in both AS mouse models and making a direct comparison. In addition, recording the USVs in adult mice or using a different protocol in newborn pups is also desirable to further characterize this intriguing phenotype in future.

The value of recording and analyzing USVs to model human autistic behaviors has been increasingly recognized, although the interpretation of data and biological meaning of USVs in rodents in relation to human behaviors is still a subject of debate [44], [53]. Reduced USVs were reported in *Foxp2* and *Nlgn4* deficiency pups [54], [55], [56], [57]. These reports supported the value of this technique because a mutation in the *FOXP2* gene was implicated in a large family with characteristic language disorder, and mutations in *NLGN4* have been found in a few cases of autism spectrum disorders in humans [58], [59]. On the other hand, similar to maternal *Ube3a* to *Gabrb3* deletion mice, increased USVs were reported in *Mecp2* deficiency pups, a mouse model of Rett syndrome, in which autistic behavior is a major feature [60]. The reason for increased USV in the Rett syndrome mouse model was also not clear. There was a suggestion that the increased USVs may be related abnormal respiratory function, a symptom commonly observed in Rett syndrome patients [43]. Interestingly, significant overlapping clinical features between AS and Rett syndrome have long been documented [61]. Thus, the increased USVs in both AS and Rett syndrome mouse models may suggest a shared pathogenesis between these two disorders. In addition, increased USVs in newborn pups was also reported in mice of BTBR T+tf/J, tuberous sclerosis gene *Tsc2* mutation, and paternal duplication of human 15q11–q13 region [62], [63], [64]. These observations suggest that increased USVs may be a common feature in mutant mice related to autism.

### A mouse model for a contiguous gene deletion form of Angelman syndrome

Although the deletion from *Ube3a* to *Gabrb3* is smaller than the typical 6-Mb deletion frequently found in human AS patients, the deleted interval in mice includes *Ube3a*, *Atp10a*, and *Gabrb3* and covers a critical region of AS deletion. Haploinsufficiency of *Atp10a* and *Gabrb3* has been suggested to account for differences in the phenotypes between patients with a point mutation in *UBE3A* and the large deletion form of AS. Thus, mice with a maternal deletion from *Ube3a* to *Gabrb3* are molecularly more similar to the AS patients with a large maternal deletion of chromosome15q11–q13. Our extensive behavioral characterization of both maternal and paternal deletion mice supports that the maternal deletion mouse is a valid model for the contiguous gene deletion form of AS. The impairments in learning and memory tasks, including contextual fear conditioning and Morris water maze, were specific to maternal, but not paternal, deletion mice. Although both light-dark and open field tests were used to evaluate anxiety related behaviors, maternal deletion mice only displayed an impairment in light-dark task but not in open field test. This may suggest a selective deficit in anxiety related behavior in maternal deletion mice. Although maternal deletion mice have significant impairment in rotarod test, there was no difference for the travel distance and speed in open field test. This was not a surprise because these mice did not have apparent motor coordination problem and abnormal gaits. The abnormal behaviors specific to the maternal deletion mice are consistent with a brain-specific, maternal expression pattern for the *Ube3a* gene. Mice used for these behavioral tests were on a relatively homogenous C57BL/6J genetic background after more than 6 generations of backcrossing to C57BL/6J. This is generally acceptable for most behavioral tests in the transgenic mutant mice although additional backcrossing may result more homogenous genetic background on C57BL/6J [65]. It should also be noted that the genetic background of the region most closely linked to the deletion at the *Ube3a* anchor is likely to be 129 SvEv and not C57BL/6J, even after backcrossing. The genetic background of the region at the *Gabrb3* anchor is C57BL/6J because the targeting construct for *Gabrb3* was produced using a C57BL/6J genomic library. The different genetic backgrounds at the targeted loci may influence the expression of genes adjacent to the targeted region. While this is technically difficult to investigate, the influence of these different genetic backgrounds on observed behavioral differences between maternal deletion mice and wild type littermate controls is probably minimal. Behavioral data from mice with a maternal *Ube3a* to *Gabrb3* deletion are mostly consistent with previous studies of *Ube3a* mutant mice, but the current study is more extensive as summarized in Table 1 [23], [25], [66]. Although mutations in the *UBE3A* gene cause typical AS in humans, it has been reported in some studies that the phenotypes such as seizures in AS patients with *UBE3A* mutations are milder than in AS patients with large deletions of 15q11–q13 [20], [21], [22]. In mice, we noticed that the frequency of observed seizure activity was not significantly different between deletion and *Ube3a* mutation mice [23]. However, these data should be interpreted with caution because the seizure phenotype, at best, is only semi-quantifiable in mice. More extensive studies with a novel design or technique are needed to compare the seizure phenotype between mice with a deletion and *Ube3a* mutation alone in order to make a meaningful conclusion. Similarly, mice with a deletion from *Ube3a* to *Gabrb3* provide a unique tool to compare other behavioral impairments that observed in mice with *Ube3a* mutation alone. However, there are potential issues in designing such comparative studies, including that mice to be tested will have to be produced from two different breeding and tests may not be carried out at the same time in parallel because of the logistic support issues. Despite of these challenges, these studies are worth to pursue because they may help to elucidate the contribution of haploinsufficiency of *Gabrb3* and *Atp10a* in the pathogenesis of AS and further shed light on the pathogenesis of human AS caused by different molecular defects.

**Table 1:** Comparison of phenotypes in Angelman syndrome mouse models with different mutations.

Features	*Ube3a* m−/p+(Jiang*)	*Ube3a* m−/p+(Miura^#^)	Deletion m−/p+ ¶
General appearance	no abnormality	No abnormality	no abnormality
Expression of *Ube3a* in brains	maternal	maternal	maternal
Hot plate test	n/a	n/a	no abnormality
Light-dark	n/a	n/a	impaired
Open field	n/a	n/a	no abnormality
Rotarod	Impaired	Impaired	impaired
PPI	n/a	n/a	no abnormalities
Fear conditioning	impaired	impaired	impaired
Morris water maze test	impaired	impaired	impaired
USVs	n/a	n/a	increased
Synaptic plasticity	hippocampal long- term potentiation defect	n/a	n/a
EEG	abnormal	abnormal	abnormal
Seizures	yes	n/a	yes

*: Targeting construct disrupted exon 2 (corresponding to human *UBE3A* exon sequence of GenBank accession No. X98022). Data are summarized from original publication[23] and subsequent studies[66], [70]

**#**: Targeting construct disrupted part of exon 15 and exon 16 (corresponding to human *UBE3A* exon sequence of GenBank accession No. AF009341)[25].

¶ : entire *Ube3a* gene was disrupted and also *Gabrb3* and *Atp10a* (this report)

n/a: not analyzed

## Materials and Methods

### Ethics statement

All protocols involving animals in this work were approved by the Institutional Animal Care and Use Committee of Baylor College of Medicine and Duke University.

### Targeting constructs for *Ube3a* and *Gabrb3* genes

Two sets of overlapping lambda phage clones containing the *Ube3a* and *Gabrb3* genes were isolated from a mouse 129/SvEv and C57BL/6J genomic library respectively. To place the *loxP* site at the *Ube3a* locus, a replacement-type targeting vector of Ube3a-pL13 was used to replace exon 2 of *Ube3a* with a cassette containing a *loxP* site, a neomycin selectable marker, and a 5′-half of the *Hprt* exon 1 and 2 minigene. To place another *loxP* site at the *Gabrb3* locus, another replacement-type targeting vector of *Gabrb3*-pG12 was constructed to replace part of exon 1 and exon 2 of *Gabrb3* with a cassette containing a *loxP* site, a puromycin selectable marker, and a 3′-half of the *Hprt* exon 3 to 9 minigene. The partial genomic and restriction enzyme map for the deleted interval between *Ube3a* and *Gabrb3*, and cassettes are shown in Figure 1A.

### Generation of mice with a chromosomal deletion from *Ube3a* to *Gabrb3*

To generate the ES cells with a deletion from *Ube3a* to *Gabrb3*, the targeting construct for Gabrb3-pG12 was linearized with *Pvu*I restriction enzyme and electroporated into 1×10^7^ AB2.2 ES cells from the 129/SvEv strain as previously described [23]. Puromycin resistant colonies were picked after 7–8 days of selection. DNA from ES cells was analyzed by mini-Southern blot hybridization using both 5′- and 3′-flanking probes for the *Gabrb3*-pG12 construct to identify clones undergoing homologous recombination. The *Gabrb3* targeted cells were expanded and used subsequently for targeting of the *Ube3a* gene. The *Ube3a*-pL13 construct linearized with *Not*I restriction was electroporated into the *Gabrb3* targeted ES cell clones with the same procedure described except that neomycin was used for selection. The *Gabrb3* and *Ube3a* double targeted ES cells were identified and subjected to electroporation with a Cre recombinase (pOG231) plasmid. HAT (hypoxanthine and thymine) selection was applied to select ES cells undergoing recombination between *loxP* sites to join the two halves of the *Hprt* minigene that conferred the HAT resistance. Abundant HAT-resistant clones were obtained from 2 out of 4 independent double-targeted ES clones, and these were further subjected to sib selection using puromycin or neomycin to determine the possible configuration of *cis* or *trans* recombination mediated by Cre recombinase. A total of 16 HAT resistant clones were sensitive to both puromycin and neomycin, indicating that the targeting was *in cis* and Cre-mediated recombination between *loxP* sites occurred as described previously [36]. ES cells with *Gabrb3* targeted, *Gabrb3* and *Ube3a* double-targeted, and a deletion from *Gabrb3* to *Ube3a* were in injected into C57 BL/6J blastocysts for germline transmission.

### Mouse breeding, genotyping, and backcrossing

Mice carrying the deletion from *Ube3a* to *Gabrb3* were backcrossed to C57BL/6J for more than 6 generations. Because of genomic imprinting in the region, a specific breeding scheme (Figure S4) was followed to produce mice for molecular studies and behavioral tests. Heterozygous males were bred to wild type C57BL/6J females to produce heterozygotes with a paternal deletion from *Ube3a* to *Gabrb3* (m+/p−) and wild type littermates (m+/p+). Heterozygous females were bred to wild type C57BL/6J males to produce heterozygotes with a maternal deletion from *Ube3a* to *Gabrb3* (m−/p+) and wild type littermates (m+/p+). Because of a possible role of maternal factors in production of ultrasonic vocalization (USV) in offspring, newborn pups used for the USV recording were produced by using male or female heterozygotes that had a deletion paternally but not maternally. Homozygotes with a deletion from *Ube3a* to *Gabrb3* (m−/p−) were produced by crossing heterozygous mice with a deletion. All experimentation and animal husbandry involving animals in this report have been conducted according to relevant national and international guidelines and under the animal protocols approved by IACUC at Duke University and Baylor College of Medicine. Genotyping was routinely doing by PCR based method using the following primer pairS: UB-R:5′ ATT AGA GGT GAT GAG GGT ATT GAG; UB-F:5′ CAT TGC AAA TCA AAG GTG TGT GGA; pL13:5′ TAC TTC CAT TTG TCA CGT CCT GCA C. PCR condition is as following: denaturation at 94°C for 45 seconds, annealing at 52°C for 45 seconds, and extension at 62°C for 45 seconds, for 35 cycles, and followed by a 5 min extension period at 72°C

### RNA isolation and RNA expression analysis

Total RNA was isolated using the Trizol method as previously described [23]. For RT-PCR analysis, DNase I-treated total RNA was reverse transcribed with Superscript RNase H^−^ reverse transcriptase (Invitrogen, Carlsbad CA) using random hexamers for *Atp10a*. PCR was carried out in 50 µl reactions with cycling conditions of denaturation at 94°C for 45 seconds, annealing at 62°C for 45 seconds, and extension at 62°C for 45 seconds, for 35 cycles. The final cycle was followed by a 5 min extension period at 72°C. Primers were ATP10AF: 5′- CTTGTCTTCAGCAGGGAAAAG-3′ and ATP10R: 5′- AATGAGTAAACTGGTGTGGGT-3′. For real-time quantitative RT-PCR using LightCycler Hydrolysis probes, the primers used were ATP10aRealT-F: 5′-ATGAAATCAACCACCTGGG-3′ and ATP10aRealT-R: 5′-TTCAGGTTGGTCTCT CCAT-3′. The probe used was 5′ FAM-CGACTTTGTCCGTCTTTGCTGCAATG-BHQ-1 3′. Primers and probe for mouse β-actin were provided by Roche (Roche applied science, Germany). Real-time quantitative PCR was carried out using a LightCycler 480 Instrument (Roche Diagnostics, Germany) and LightCycler 480 RNA Master Hydrolysis Probes following manufacturer's recommendations. Two µl of the total RNA sample was used in a 20 µl PCR reaction containing 1x LightCycler 480 RNA master mix with an appropriate concentration of primers and probe. All samples and experiments were run in triplicate. Each PCR run also included 3 samples without RNA template as negative controls as well as a consistent triplicate calibrator comprising a mixture of multiple mouse cerebellum RNA as template. Mouse β-actin RNA expression was used as an internal reference to normalize the quantity of RNA input across samples for quantification analysis.

### Western blot analysis

Mouse brain tissues were homogenized in NP40/SDS buffer (1% Nonidet P-40, 0.01% SDS, 0.1 M Tris-HCl, pH 7.2, and complete Protease Inhibitor Cocktail Tablet; Roche Applied Sciences, Indianapolis, IN) on ice with a pellet pestle. Following polyacryl amide gel electrophoresis, proteins were transferred to nitrocellulose membrane (Bio-Rad; Hercules, CA) at 4°C overnight. Incubations with antibodies were performed in TPBS-milk at 4°C overnight as follows: rabbit anti-human E6-AP diluted 1∶1,000 (BL447; Bethyl Labs, Montgomery, TX); mouse Gabrb3 diluted 1∶500 (LC-43101, Santa Cruz Biotec, Santan Cruz CA); and anti-mouse actin diluted 1∶500 (SC-1606, Santa Cruz Biotec. Santa Cruz, CA). The signals were detected by enhanced chemiluminescence (ECL, Amersham Life Science, Newark, NJ). The Image J software was used for quantification.

### Chronic electroencephalographic (EEG) recordings

Paternal and maternal deletion mice and wild-type littermates (aged 3–4 months) were implanted for chronic EEG monitoring. Mice were anesthetized with Avertin (1.25% tribromoethanol/amyl alcohol solution, i.p.) using a dose of 0.02 ml/g. Teflon-coated silver wire electrodes (0.005 inch diameter) attached to a microminiature connector were implanted bilaterally into the subdural space over temporal, parietal, and occipital cortices. Digital EEG activity was monitored daily for up to two weeks during prolonged overnight and random 2 hr sample recordings (Stellate Systems, Harmonie software version 6.1b; Montreal Quebec, Canada). A digital video camera was used to simultaneously monitor behavior during the EEG recording periods. All recordings were carried out at least 24 hours after surgery on mice freely moving in the test cage.

### Mouse behavioral testing

A battery of behavioral test was performed using a protocol previously described and used in behavioral core facilities at Baylor College of Medicine [23], [67], [68]. These tests include open field, light dark, rotarod, prepluse inhibition (PPI), fear conditioning, Morris water maze, hot plate, and ultrasonic vocalization recording (USVs). Mice were tested in the order listed except USVs for which a different set of mice were used. Mice were rested for one week between each test except only 1–3 days between open field and light dark and two to three weeks between fear conditioning and Morris water maze. The procedure and sequence of the tests, except of USVs recording, were previously described and validated [67], [68], [69]. A different set of newborn pups was used for the USVs recording and these pups were not be used for other behavioral tests. The protocol for USVs recording was based on a previous report with minor modifications [45]. A detailed description of each protocol is included in the supplementary data, Method S1.

### Statistical analysis

Raw data from all behavioral tests were documented and tabulated in Microsoft excel. Statistical analyses were performed using Origin 8 software (http://www.originlab.com/) and SAS (www.sas.com) packages. For most behavioral data, analyses were performed by using one-way ANOVA. Two-way and three-way ANOVA with repeated measure was used as indicated. Post hoc analysis was also performed as indicated.

## Supporting Information

Figure S1 Open field and hot plate test. A, B, C: Results of open filed test. Travel distance per block for m−/p+ (A) and m+/p− (B), as well as total travel distance (C) of m−/p+ and m+/p− mice in open field test. D. Results of hot plate test.
(0.02 MB PDF)

Figure S2 Results of Pre-pulse inhibition in mice. No difference in maximal startle response between m−/p+ mice and their m+/p+ littermates or between m+/p− mice and their m+/p+ littermates.
(0.01 MB PDF)

Figure S3 The production of USVs of maternal deletion pups in different days and beddings.
(0.02 MB PDF)

Figure S4 Breeding of *Ube3a* and *Gabrb3* deletion mice. Only F1 and F2 are diagrammed in figure. Mice and pups used for the experiments were from >F6 of backcrossing to C57BL/6J.
(0.02 MB PDF)

Method S1 Protocols for behavioral testing.
(0.04 MB DOC)

Video S1 EEG recording video.
(16.39 MB ZIP)
