# Supplementary figures and images for: Altered Ultrasonic Vocalization and Impaired Learning and Memory in Angelman Syndrome Mouse Model with a Large Maternal Deletion from Ube3a to Gabrb3
Source: PLoS One. 2010 Aug 20;5(8):e12278. doi: 10.1371/journal.pone.0012278 (PMC2924885; doi:10.1371/journal.pone.0012278)

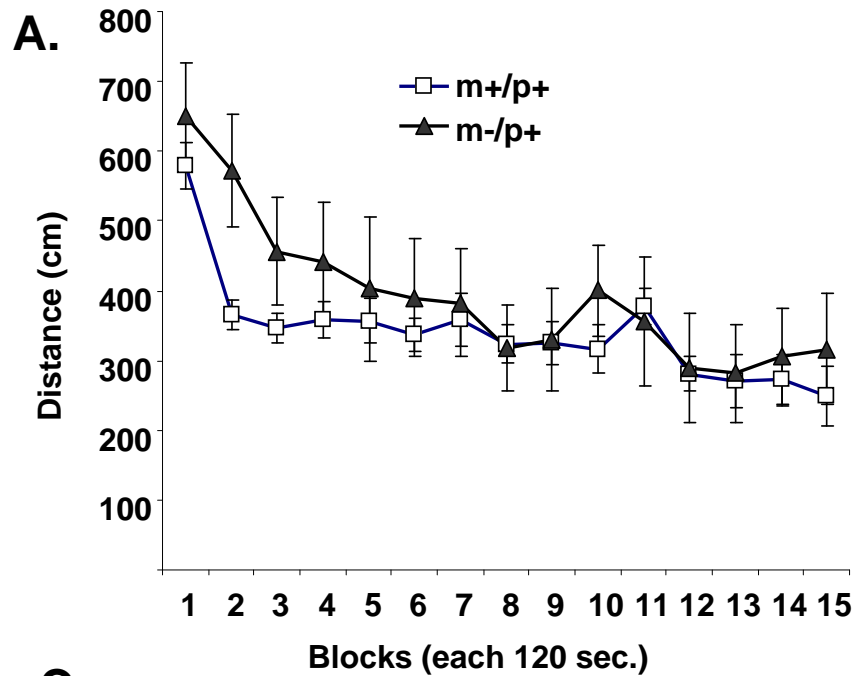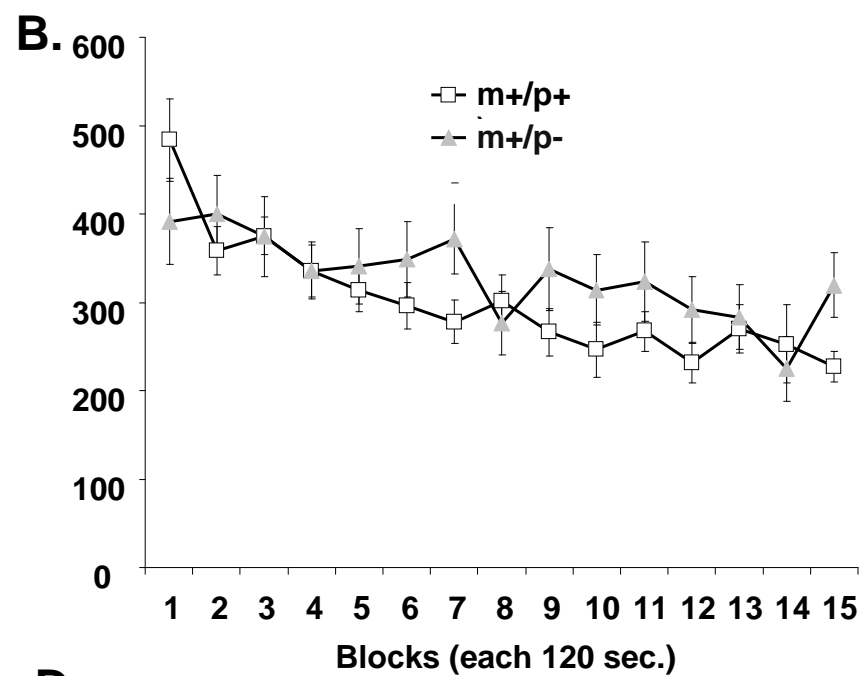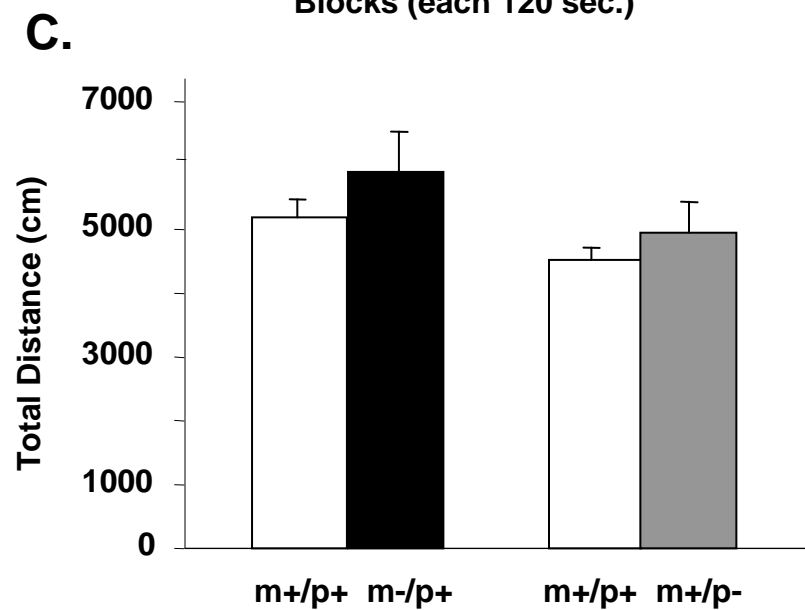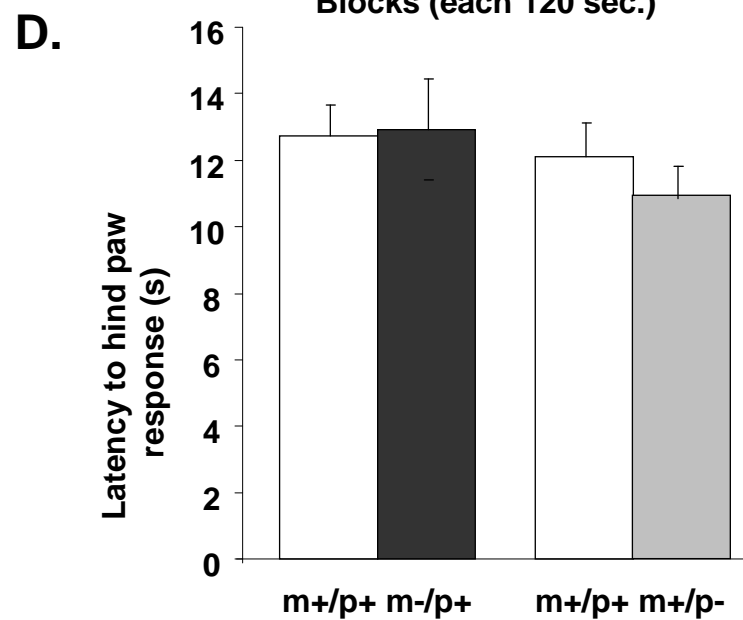

Supplement: Figure S1 — Open field and hot plate test. A, B, C: Results of open filed test. Travel distance per block for m−/p+ (A) and m+/p− (B), as well as total travel distance (C) of m−/p+ and m+/p− mice in open field test. D. Results of hot plate test. (0.02 MB PDF) [file pone.0012278.s001.pdf]

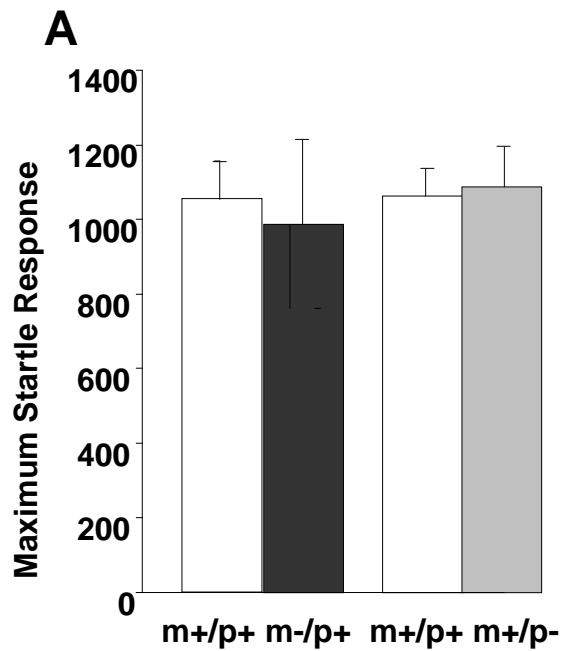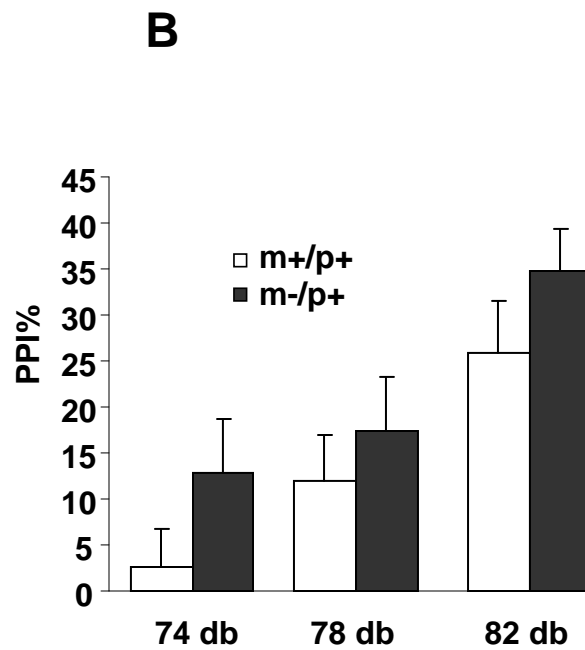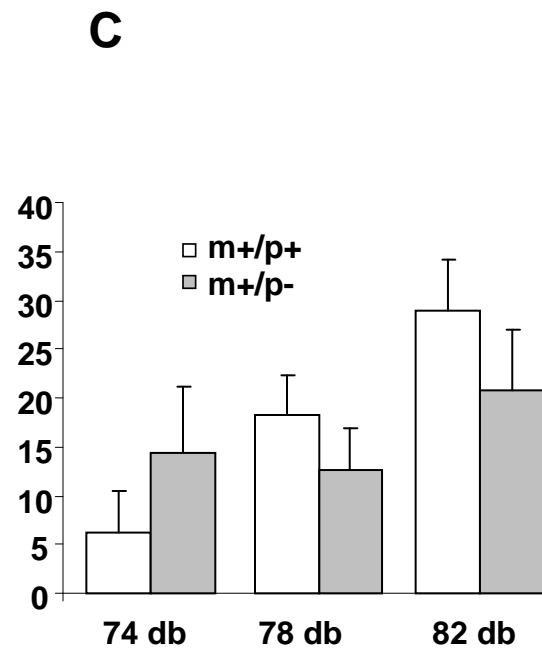

Supplement: Figure S2 — Results of Pre-pulse inhibition in mice. No difference in maximal startle response between m−/p+ mice and their m+/p+ littermates or between m+/p− mice and their m+/p+ littermates. (0.01 MB PDF) [file pone.0012278.s002.pdf]

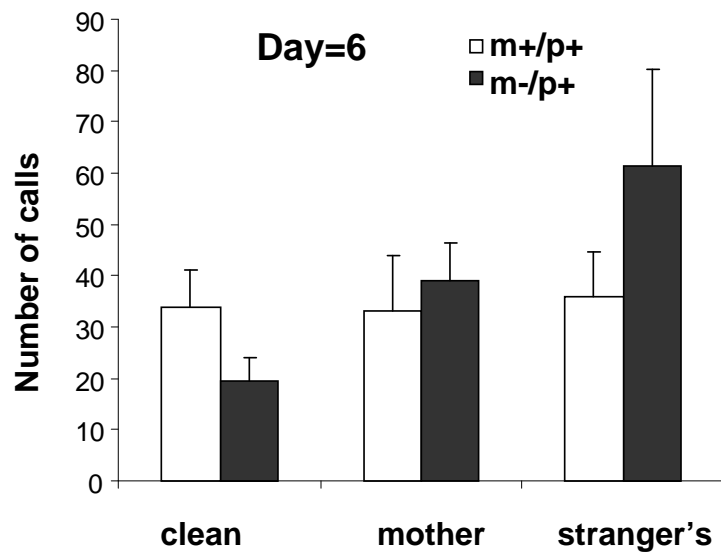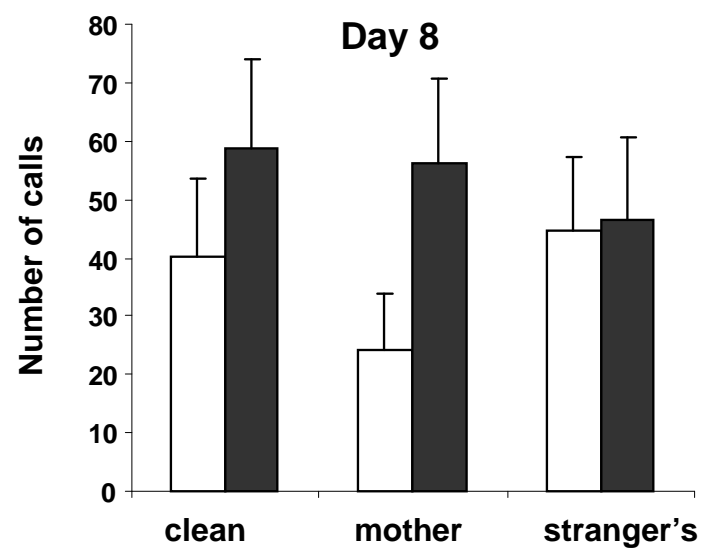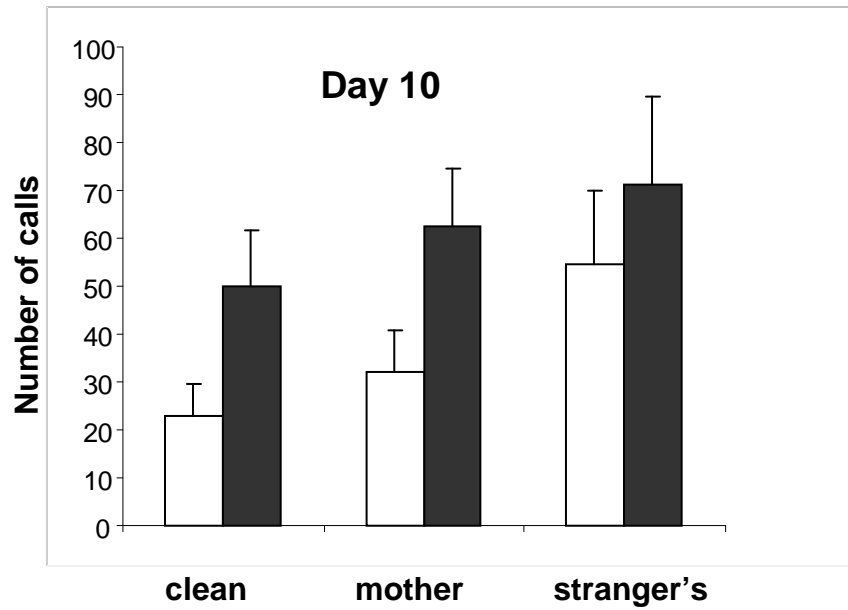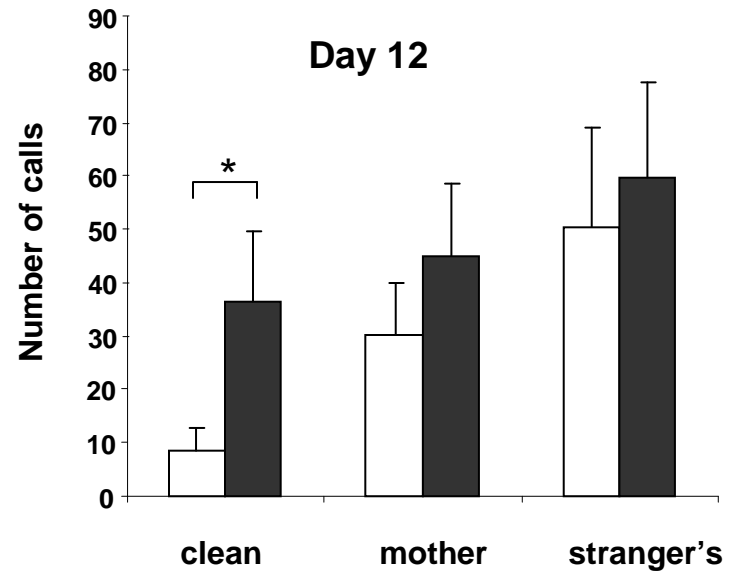

Supplement: Figure S3 — The production of USVs of maternal deletion pups in different days and beddings. (0.02 MB PDF) [file pone.0012278.s003.pdf]

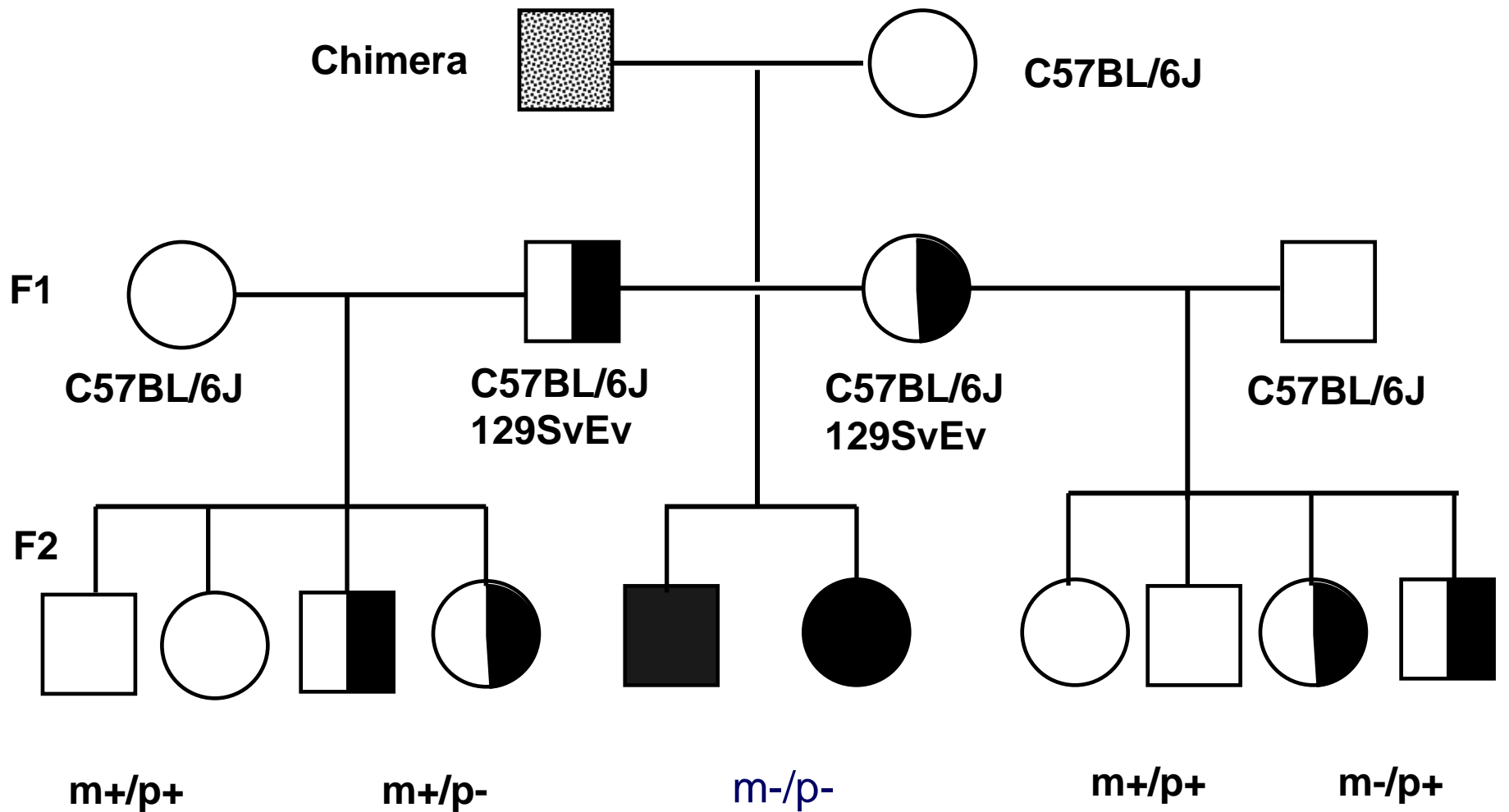

>F6 mice were used for experiments

>F6 mice were used for experiments

Supplement: Figure S4 — Breeding of Ube3a and Gabrb3 deletion mice. Only F1 and F2 are diagrammed in figure. Mice and pups used for the experiments were from >F6 of backcrossing to C57BL/6J. (0.02 MB PDF) [file pone.0012278.s004.pdf]
