# Supplementary material for: Altered Ultrasonic Vocalization and Impaired Learning and Memory in Angelman Syndrome Mouse Model with a Large Maternal Deletion from Ube3a to Gabrb3
Source: PLoS One. 2010 Aug 20;5(8):e12278. doi: 10.1371/journal.pone.0012278 (PMC2924885; doi:10.1371/journal.pone.0012278)
Supplement: Method S1 — Protocols for behavioral testing. (0.04 MB DOC) [file pone.0012278.s005.doc]

**Supplemental data-protocol for behavioral tests**

**Locomotor activity in the open field**
 Locomotor activity was evaluated by placing a mouse into the center of a clear Plexiglas (40x40x30 cm) open-field arena and allowing the mouse to explore for 30 min. Bright overhead lighting was approximately 500 lux inside the arenas, while white noise was present at approximately 60 dB. Activity in the open-field was quantified by a computer-operated Digital optical animal activity system (Accuscan Electronics, Columbus, OH). Total distance (locomotor activity), movement time (in seconds), movement speed (cm/s), and center distance (the distance traveled in the center of the arena) were recorded. The center distance was divided by the total distance to obtain a center distance–total distance ratio. The center distance–total distance ratio can be used as an index of anxiety-related responses. Data were collected in 2 min intervals over the 30 min test session. Analysis was performed by one way ANOVA or two-way (genotype x block) ANOVA with repeated measure.

**Light–dark exploration**
 Mice were then tested in the light–dark exploration test, which consists of a polypropylene chamber (44x21x21 cm) unequally divided into two chambers by a black partition containing a small opening. The large chamber is open and brightly illuminated (800 lx), while the small chamber is closed and dark. White noise is present in the room at
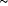
55 dB in the test chamber. Mice were placed into the illuminated side and allowed to move freely between the two chambers for 10 min. Latency to enter and duration and number of entries to light and dark compartments were determined using a hand-held computer (Psion Workabout mx, Psion Teklogix) together with the OBSERVER® program (Noldus Information Technologies, Leesburg, VA). An entry was defined as the mouse placing all four feet into the zone. Analysis was performed by using one-way ANOVA.

**Startle and prepulse inhibition of the startle**
 Mice were tested for prepulse inhibition of acoustic startle responses using the SR-Lab System (San Diego Instruments, San Diego, CA, USA), as previously described. A test session began by placing a mouse in the Plexiglas cylinder where it was left undisturbed for 5 min. A test session consisted of eight trial types. One trial type was a 40 ms, 120 dB sound burst used as the startle stimulus. There were three different acoustic prepulse plus acoustic startle stimulus trial types. The prepulse sound was presented 100 ms before the startle stimulus. The 20 ms prepulse sounds were at 74, 78, and 82 db. Trials with prepulses alone were used to verify that mice did not startle to the prepulse sounds. Finally, there were trials where no stimulus was presented to measure baseline movement in the cylinders. Six blocks of the eight trial types were presented in pseudorandom order such that each trial type was presented once within a block of eight trials. The average inter-trial interval was 15 s (ranged from 10 to 20 s). The startle response was recorded for 65 ms (measuring the response every 1 ms) starting with the onset of the startle stimulus. The background noise level in each chamber was
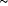
70 dB. The maximum startle amplitude recorded during the 65 ms sampling window was used as the dependent variable. Percent prepulse inhibition of the startle response was calculated as 100 - [(startle response on acoustic prepulse plus startle stimulus trials / startle response alone trials) X 100]. Data analysis was performed by using two-way ANOVA (genotype x dB).

**Hot plate test**

The hot-plate test was used to evaluate sensitivity to a painful stimulus. Mice were placed on a 55.0°C (±0.3) hot-plate (Columbus Instruments, Columbus, OH), and the latency to the first hind-paw response was recorded. The hind-paw response was either a foot shake or a paw lick. Data analysis was performed by using one-way ANOVA.

**Rotarod test**
 Mice were placed on a rotating rod (model 7650 Rota-rod, Ugo Basile, Collegeville, PA) that accelerated from 4 to 40 rpm. For two consecutive days, four trials were performed per day with 45–60 min interval between trials. The maximum duration of each trial was 5 min. The time that the mice fell off the rod was recorded. Data analysis was performed by using two-way ANOVA (genotype x trials) with repeated measures.

**Fear conditioning**
 The test chamber (26 cm x 22 cm x18 cm high) had clear Plexiglas sides and a grid floor that was used to deliver a mild foot shock (Actimetrics chamber system, Med Associates, St. Albans, VT). The chamber was placed inside a sound-attenuated chamber (Med Associates, internal dimensions: 56 cm x 38 cm x 36 cm) that had a window through which mice could be observed without disturbance. On the training day, mice were placed into the test chamber and allowed to explore for 2 min. The conditioned stimulus (CS) (a white noise 80 dB sound) was presented for 30 s and followed immediately by a mild foot shock (2 s, 0.7 mA) that served as the unconditioned stimulus (US). After 2 min, the mice received a second CS–US pairing. The Freeze Frame monitor system (San Diego Instruments, San Diego CA) was used to control the timing of CS and US presentations and to record freezing behavior. During the conditioning procedure, responses to the foot shock - typically run, jump, or vocalize - were also recorded.

Mice were tested for contextual and cued fear conditioning 24 h after conditioning. For the context test, mice were placed back into the original test chamber for 5 min and freezing behavior was recorded. One to two hours later, mice were tested for responses to the auditory CS in a new environment. For the CS test, black Plexiglas inserts were placed over the sides and floor of the chamber to alter the shape, texture and color of the chamber. Vanilla extract was placed in the chamber behind the insert to alter the odor. Transfer cages were altered (paper towels instead of bedding) and red house lights replaced the normal white house lights. Mice were placed into this new chamber and freezing was recorded for 3 min during this ‘pre-CS’ phase. The auditory CS was then presented for another 3 min and freezing was recorded as described. Data for the CS test were calculated as the percent freezing during the CS minus percent freezing in the pre-CS phase. Data analysis was performed by using one-way ANOVA.

**Morris water maze task**

Mice were trained in the Morris water maze task to locate a hidden escape platform in a circular pool (1.38 m diameter) of water. Each mouse was given eight trials a day, in two blocks of four trials separated by at least 2 hours for four consecutive days, for a total of 32 trials. The time taken to locate the escape platform (escape latency) and the distance traveled were determined using the Ethovision tracking system (Noldus Information Technologies, Leesburg, VA). After trial 32, each animal was given a probe trial, during which the platform was removed and each animal was allowed 60 s to search the pool. The amount of time that each animal spent in each quadrant was recorded (quadrant search time). The number of times a subject crossed the exact location of the platform during training was determined, and compared with crossings of the equivalent location in each of the other quadrants (platform crossing). The labeling for each quadrant and position for hidden platform is diagramed in Figure 6G. Escape latency data were analyzed by two-way (genotype x trail) ANOVA with repeated measure. The platform crossing data were analyzed by using one-way ANOVA followed by the post hoc analysis.

**Recording of ultrasonic vocalizations (USVs)**

We recorded ultrasonic vocalizations (USVs) in a sound-attenuated chamber using the UltraVox version 2 system (Noldus Information Technologies, Leesburg, VA). A bat detector was positioned approximately 10 cm above the subject and set to a frequency of 70±5 kHz. The UltraVox software was set to distinguish between USVs with a minimum duration (on time) of 5 msec and separated by at least 30msec (off time). Only first litters with four to six pups were used for recording. Because of concern that mice who inherited a maternal deletion may have abnormal behavior and affect the maternal pup interaction, only mice who inherited deletion paternally were used for breeding and producing the pups for recording USVs. Recording was done for pups at age 6, 8, 10, and 12 days when they were separated from mother. Litters were removed from the parental cage and kept in a new cage kept at 32-35°C with a heating pad. Then the pups, one at a time, were placed in a 200 ml glass beaker with bedding in the following order: 1) clean supply, 2) from home cage (mother’s), and 3) other breeding cage (stranger’s). The ultrasonic vocalizations during a 5 min period at room temperature 21^o^C were recorded. Testing usually started at 9 AM and finished before 3 pm during the testing days. The pups were returned to home cages with mothers immediately after each recording. The pups were not separated from the mother for more than 30 min at a time and there was at least one hour rest time before the next test. On days 10 and 12, a fourth test was added to the end of day- USVs were recorded at 4-6 ^o^C using clean bedding in a container placed inside another ice-water filled container. Pups were marked with a non-invasive pen after the first test and repeated as needed. All pups were genotyped after completion of all recording at day 13 and gender was recorded at the same time. Data analyses were performed by using one-way ANOVA.
